# Reliability and validity of the Japanese version of the Resilience Scale and its short version

**DOI:** 10.1186/1756-0500-3-310

**Published:** 2010-11-17

**Authors:** Daisuke Nishi, Ritei Uehara, Maki Kondo, Yutaka Matsuoka

**Affiliations:** 1 Department of Psychiatry and Clinical Research Institute, National Disaster Medical Center, Tokyo, Japan; 2 CREST, Japan Science and Technology Agency, Tokyo, Japan; 3 National Institute of Mental Health, National Center of Neurology and Psychiatry, Tokyo, Japan; 4 Department of Public Health, Jichi Medical University, Tochigi, Japan; 5 Aoyama Counseling Center, International University of Health and Welfare Graduate School, Tokyo, Japan

## Abstract

**Background:**

The clinical relevance of resilience has received considerable attention in recent years. The aim of this study is to demonstrate the reliability and validity of the Japanese version of the Resilience Scale (RS) and short version of the RS (RS-14).

**Findings:**

The original English version of RS was translated to Japanese and the Japanese version was confirmed by back-translation. Participants were 430 nursing and university psychology students. The RS, Center for Epidemiologic Studies Depression Scale (CES-D), Rosenberg Self-Esteem Scale (RSES), Social Support Questionnaire (SSQ), Perceived Stress Scale (PSS), and Sheehan Disability Scale (SDS) were administered. Internal consistency, convergent validity and factor loadings were assessed at initial assessment. Test-retest reliability was assessed using data collected from 107 students at 3 months after baseline. Mean score on the RS was 111.19. Cronbach's alpha coefficients for the RS and RS-14 were 0.90 and 0.88, respectively. The test-retest correlation coefficients for the RS and RS-14 were 0.83 and 0.84, respectively. Both the RS and RS-14 were negatively correlated with the CES-D and SDS, and positively correlated with the RSES, SSQ and PSS (all p < 0.05), although the correlation between the RS and CES-D was somewhat lower than that in previous studies. Factor analyses indicated a one-factor solution for RS-14, but as for RS, the result was not consistent with previous studies.

**Conclusions:**

This study demonstrates that the Japanese version of RS has psychometric properties with high degrees of internal consistency, high test-retest reliability, and relatively low concurrent validity. RS-14 was equivalent to the RS in internal consistency, test-retest reliability, and concurrent validity. Low scores on the RS, a positive correlation between the RS and perceived stress, and a relatively low correlation between the RS and depressive symptoms in this study suggest that validity of the Japanese version of the RS might be relatively low compared with the original English version.

## Background

Resilience is a multidimensional concept variously defined as a personal trait protective for mental disorders and a dynamic process of adaptation to challenging life conditions [1,2], and it has generally been viewed as a stress coping ability in the face of adversity. Various positive features of mental health such as strong self-esteem and self-efficacy have been shown as the characteristics of resilient people [3].

Clinical significance of resilience has received some considerable attention. For example, coping self-efficacy, which is the belief in one's own ability to manage posttraumatic recovery demands, was shown to be an important predictor for psychological adjustment to a variety of traumas [4].

In addition, the previous study showed that depressed patients felt the presence of positive features of mental health such as optimism, vigor and self-confidence were a better indicator of remission rather than the absence of the depressive symptoms [5]. Recently, resilience has been defined as a potentially modifiable factor [6] and might be improved through intervention [7].

Although a number of scales measuring resilience have been developed, a recent review [8] determined that Wagnild and Young's Resilience Scale (RS) [9] was currently the best instrument to study resilience in adolescent populations because RS has been used successfully in many studies. The RS is a 25-item self-report questionnaire first developed in 1993, and a short version consisting of 14 items (RS-14) has recently developed to reduce participant burden by Wagnild [10]. Internal consistency and concurrent validity of the original RS and RS-14 were shown to be good, and RS was later joined by Russian [11], Spanish [12] and Swedish version [13]. However, Japanese version of RS has not been developed.

The aim of the present study was to demonstrate and report on the reliability and validity of the Japanese-language version of the RS and RS-14, developed by the authors in with the cooperation of the original developers.

## Methods

### 2.1 Subjects and Procedures

The study protocol was approved by the Institutional Review Board and Ethics Committee of the National Disaster Medical Center (NDMC), Tokyo. Participants in the present study were students recruited from a nursing college and university students taking a psychology course. After permission for access to the students was obtained from the head of the nursing college and the course leaders of the university psychology laboratory, the first author (DN) and third author (MK) visited the classrooms, where the questionnaires were administered. At the nursing college, the questionnaires were collected only from students who agreed to participate. At the university, the questionnaires were collected from all students, and those who did not wish to participate in the study checked a box to withdraw their participation. Participants were 229 of the 258 nursing college students and 268 of the 275 university psychology laboratory students. A subsample of the nursing college students were retested on the RS 3 months after initial assessment. Internal consistency, convergent validity and factor loadings were assessed at the initial assessment, and test-retest reliability was assessed at the second assessment. Because this study was conducted anonymously, postal code and nicknames which participants gave themselves at initial assessment were identified to examine test-retest reliability.

### 2.2 The Resilience Scale (RS)

The RS measures the degree of individual resilience. It consists of 25 items, and the degree of stress perceived for each item is rated on 7-point Likert scales (range, 25-175) [9]. The RS-14 is the short version of the RS, which is strongly correlated with the RS. It consists of 14 of the RS items: 2, 6, 7, 8, 9, 10, 13, 14, 15, 16, 17, 18, 21 and 23 [10].

Internal consistency and concurrent validity of the RS was shown to be good [8]. Regarding the scale's factor structure, Wagnild and Young suggested that a two-factor solution was interpretable. The factor labeled Personal Competence consists of 17 items, thought to measure self-reliance, independence, determination, invincibility, mastery, resourcefulness and perseverance. The factor labeled Acceptance of Self and Life consists of 8 items, thought to measure adaptability, balance, flexibility and a balanced perspective on life.

With the original authors' permission, we translated the English RS into Japanese. We followed the standard procedure of back-translation to ensure fidelity across language versions. The first author (DN) translated the English RS into Japanese. This preliminary Japanese RS was back translated into English by an independent translator. The back-translated version was examined by Dr. Gail M. Wagnild, one of the original developers of the RS. The first author then corrected the Japanese translation accordingly. This process was repeated until both the original authors and the current authors agreed that the original and back-translated versions closely matched.

### 2.3 Other Measures

#### The Center for Epidemiologic Studies Depression (CES-D)

The CES-D, which is one of the most widely used scales to assess depressive symptoms, measures the level of depressive symptoms in the past one week [14]. The test-retest reliability and concurrent validity of the Japanese version of the CES-D has been thoroughly documented [15]. It consists of 20 items, and the degree of distress for each item is rated on a 4-point scale (range, 0-60). RS was shown to be negatively correlated with depression [10].

#### Rosenberg Self-Esteem Scale (RSES)

The RSES assesses the overall sense of being capable, feeling worthwhile, and competence [16]. Internal consistency and factor validity of the Japanese version of the RSES was shown to be high [17]. It consists of 10 items, and the degree of self-esteem for each item is rated on a 5-point Likert scale (range, 10-50). RS was shown to be positively correlated with self-esteem [9].

#### Social Support Questionnaire (SSQ)

The SSQ assesses the perceived availability of and satisfaction with social support, which is usually defined as the existence or availability of people on whom we can rely [18,19]. Internal consistency, factor validity and construct validity of the Japanese version of the SSQ was shown to be high [20]. The short version of the SSQ consists of 12 items. Six of the items measure perceived number of social supports, and the other 6 items measure satisfaction with social support; each item is rated on a 6-point Likert scale. The average scores for the two domains are calculated. RS was shown to be positively correlated with inter-personal support [10].

#### Perceived Stress Scale (PSS)

The PSS measures the global level of perceived stress, dealing with the degree to which situations in one's life are appraised as stressful as opposed to the presence of particular stressors [21]. Higher scores on the PSS equate with more perceived stress. The Japanese version of the PSS has high internal consistency and factor validity [22]. It consists of 14 items, and the degree of stress for each item is rated on a 5-point Likert scale (range, 0-56). RS was shown to be negatively correlated with perceived stress [9].

#### Sheehan Disability Scale (SDS)

The SDS measures individual disability in three domains: work or school, social and family life, and home responsibilities [23]. Internal consistency, test-retest validity and concurrent validity of the Japanese version of the SDS was shown to be good [24]. The degree of disability for each item is rated on an 11-point Likert scale (range, 0-30). The Connor-Davidson Resilience Scale (CD-RISC), which is another well-known scale to measure resilience, was shown to be negatively correlated with SDS [25].

In addition, the assessments collected information on age, gender, and past history of psychiatric illness.

### 2.4 Statistical Analyses

Cronbach's alpha coefficient, the test-retest correlation coefficient, and the correlations between the RS and other measures were established by calculating Pearson's correlation coefficients. Factor analysis was conducted by using data at initial assessment. All statistical analyses used two-tailed tests. For all statistical evaluations, p values less than 0.05 were considered indicative of significant differences. All data analyses were performed using the statistical software package SPSS, version 14.0J for Windows (SPSS Japan Inc., Tokyo, Japan).

## Results

Participants were 229 of the 258 nursing college students and 268 of the 275 university psychology laboratory students. Of these 497 participants, at least one item was missing in the response of 67 participants. Thus, data from 430 participants were analyzed. Sixty seven participants who dropped out of the study had more number of social supports and more satisfaction with social support (*p *< .05). They did not differ significantly from 430 participants in terms of age, sex, RS, CES-D, RSES, PSS and SDS. There were 117 retest samples obtained from the nursing college.

Of the 430 participants, 345 (80.2%) were women and 417 (97.0%) were not married. Median age was 19.0 years (range, 18-51), and 24 (5.6%) self-reported a past history of psychiatric illness.

Mean score on the RS was 111.19. The items are presented in Table 1. The overall Cronbach's alpha coefficient of the RS was 0.90, and that of the RS-14 was 0.88. The test-retest correlation coefficient of the RS was 0.83, and that of the RS-14 was 0.84. The initial assessment RS scores of the subsample who were later retested were significantly higher (mean = 116.0, SD = 20.78, p < 0.01) than those of the rest of the total sample who dropped out of the follow-up assessment (mean = 109.4, SD = 18.68).

**Table 1:** Mean scores and standard deviations of the Japanese version of the Resilience Scale (N = 430)

Item description	Mean	SD
1. Follow through with plans	4.07	1.43
2. Manage one way or other	5.28	1.01
3. Able to depend on self more than anyone4.421.32	4.42	1.32
4. Keeping interested in things is important	5.65	1.07
5. I can be on my own if I have to	4.84	1.50
6. Proud that I have accomplished things	4.31	1.53
7. I take things in stride	4.45	1.40
8. I am friends with myself	4.50	1.45
9. I can handle many things at a time	3.36	1.48
10. I am determined	3.68	1.60
11. Seldom wonder what the point of it is	4.17	1.72
12. I take things one day at a time	4.31	1.45
13. I can get through difficult times	4.70	1.36
14. I have self-discipline	4.64	1.37
15. I keep interested in things	3.66	1.52
16. Find something to laugh about	4.85	1.43
17. My belief in myself gets me through	4.46	1.34
18. In an emergency, people can rely on me	3.88	1.43
19. Look at a situation in a number of ways	4.33	1.38
20. I make myself do things	4.81	1.35
21. My life has meaning	4.83	1.52
22. I do not dwell on things	4.03	1.79
23. I can find my way out of it	4.22	1.29
24. Have energy to do what I have to	4.85	1.28
25. It's okay if people don't like me	4.90	1.75
		
RS-14 (14 items)	63.78	13.03
RS (25 items)	111.19	19.47

RS-14 includes items 2, 6, 7, 8, 9, 10, 13, 14, 15, 16, 17, 18, 21, and 23.

Indicators of concurrent validity of the RS and RS-14 are shown in Table 2. Both the RS and RS-14 were negatively correlated with the CES-D and SDS, and positively correlated with the RSES, SSQ and PSS.

**Table 2:** Correlations between the Resilience Scale (RS) total score and other measures

		RS (25 items)		RS-14 (14 items)	
	**Mean (SD)**	**Correlation** **Coefficients**	**p value**	**Correlation** **Coefficients**	**p value**

CES-D	16.9 (7.9)	-0.30	< 0.01	-0.28	< 0.01
RSES	32.4 (3.8)	0.27	< 0.01	0.28	< 0.01
SSQ					
Number	4.1 (1.9)	0.34	< 0.01	0.38	< 0.01
Satisfaction	4.8 (1.2)	0.11	0.02	0.12	0.01
PSS	23.8 (7.3)	0.15	< 0.01	0.18	< 0.01
SDS	8.2 (6.9)	-0.32	< 0.01	-0.32	< 0.01

Abbreviations: CES-D, Center for Epidemiologic Studies Depression Scale; RSES, Rosenberg Self-Esteem Scale; SSQ, Social Support Questionnaire; PSS, Perceived Stress Scale; SDS, Sheehan Disability Scale

Factor analyses with an eigenvalues greater than 1.0 were used to explore dimensions in the RS. From a principal component extraction with varimax rotation, six factors were emerged. However, the six-factor solution considering only those factor loadings greater than 0.40 contained secondary loadings and difficulties in interpreting the factors (Additional file 1). Similarly, the two-, three-, four-, and five-factor solutions were also ambiguous. One-factor solution accounted for only 31.5% of the total variance.

The 14 items in the RS-14 were entered into principal component analysis according to the previous study [10]. All items loaded onto the first component, and factor loadings were greater than 0.49. One-factor solution accounted for 39.4% of the total variance.

## Discussion

This study demonstrates that internal consistency and test-retest reliability of the Japanese version of the RS are high, and its concurrent validity is relatively low. Moreover, the Japanese version of the short form RS-14 was equivalent to the RS in internal consistency, test-retest reliability, and concurrent validity.

Connor and Davidson pointed out that scales measuring resilience could be tools not only to identify resilient characteristics but also to assess response to therapy, because an individual tends to become engaged in more adaptive pursuits and their problems tend to diminish in focusing strengths and positive attributes [25]. In this view, the Japanese version of RS and RS-14 could be applied to psychological interventions in the future.

As expected, resilience as measured by the RS was positively correlated with self-esteem and social support, and negatively correlated with depressive symptoms and individual disability, although the correlation between resilience and depressive symptoms was relatively lower than those in previous studies [9,11,12]. Self-esteem has been viewed as one of the components of resilience [3]. Also, resilient persons are thought to have the ability to extract and enhance social support from others [6], and to be less vulnerable to depression and individual disability. Thus, these results are consistent with the concept of resilience.

Interestingly, resilience as measured by the RS was positively correlated with perceived stress as measured by the PSS. Although the relationship between the RS and PSS has not been examined previously, RS was shown to be negatively correlated with perceived stress [9] and CD-RISC was also shown to be negatively correlated with the PSS [25]. Possible explanation is that concurrent validity of the Japanese version of the PSS has not been fully established [22]. Further studies might be needed to elucidate the relationship between resilience and perceived stress.

The mean RS score in the present study was 111.19, which is much lower than that obtained in previous studies [9,10,12,26]. Wagnild and Young showed that scores greater than 145 indicate moderately high to high resilience, scores between 121-145 indicate moderately-low to moderate levels of resilience, and scores of 120 and below indicate low resilience [9]. In fact, the mean score of patients with bipolar disorder in the United States has been shown to be 120.75 [27]. However, it is highly unlikely that the majority of the participants in the present study have such low resilience levels.

Regarding this issue, Iwata et al. [28] pointed out that Japanese respondents have a general tendency to suppress the expression of positive affect, and therefore report a significantly lower level of positive affect on the CES-D than non-Japanese, while their negative symptom scores are comparable to those of other ethnic groups [29]. This Japanese tendency may help explain the extremely low RS scores, the positive correlation between the RS and PSS, and the relatively low correlation between the RS and depressive symptoms found in the present study, which may limit the validity of this Japanese version of the RS.

The result of factor analyses was not consistent with previous studies. One possible reason, and a limitation in this study, is the sampling procedures in this study. The participants were all students who were mostly young, single women, with no past history of psychiatric illness, and not randomly selected. Especially, previous studies pointed out that age was important for resilience [9,13]. The sampling procedure in this study could influence the results which were different from other previous studies. On the other hand, principal component analysis indicated a one-factor solution for the Japanese version of the RS-14. This result was consistent with the original versions of the RS-14 [10]. At present, it may be better for researchers and clinicians to use RS-14 in Japanese adolescents, in terms of interpretable factor structure, and equivalent reliability and validity to those of RS.

The present study has some limitations. As mentioned above, one of the major limitations is the sampling procedure. Not only factor structure but also the reliability and validity of the Japanese RS and RS-14 in different populations should be further studied. Also, the baseline RS scores of the subsample who were retested were higher than those of the participants who did not participate in the second assessment. This might have affected the test-retest reliability in the present study. Moreover, divergent validity was not examined in the present study. It would also be desirable to examine correlations between the RS and conceptually different measures to ensure that the RS is specific enough to evaluate resilience, even though resilience is a multidimensional construct and will therefore be related to many other measures.

## Conclusions

This study demonstrates that both the Japanese RS and RS-14 have psychometric properties with high degrees of internal consistency, high test-retest reliability, and relatively low concurrent validity. Low scores on the RS, a positive correlation between the RS and perceived stress, and a relatively low correlation between the RS and depressive symptoms in this study suggest that validity of the Japanese version of the RS might be relatively low compared with the original English version.

## Competing interests

This work was also supported by grants from the Foundation for Total Health Promotion, Toray Industries Inc. and Japan Science and Technology Agency, CREST. Funding sources did not play any other role. The authors did not have any competing interests in this work.

## Authors' contributions

DN conceived of the study, carried out investigation, analyzed data, and drafted the manuscript. RU participated in the design of the study. MK participated in the design of the study, and carried out investigation. YM participated in the design of the study. RU, MK and YM revised the manuscript critically for important intellectual content. All authors read and approved the final manuscript.

## Supplementary Material

Additional file 1 **Factor loadings from factor analysis**. The result of factor analyses using principal component extraction with varimax rotation. Click here for file
