# Supplementary material for: Reliability and validity of the Japanese version of the Resilience Scale and its short version
Source: BMC Res Notes. 2010 Nov 17;3:310. doi: 10.1186/1756-0500-3-310 (PMC2993730; doi:10.1186/1756-0500-3-310)
Supplement: Additional file 1 — Factor loadings from factor analysis. The result of factor analyses using principal component extraction with varimax rotation. [file 1756-0500-3-310-S1.DOC]

Additional file 1

Title: Factor loadings from factor analysis

Description: The result of factor analyses using principal component extraction with varimax rotation.

|  | Factor (Eigenvalue) | | | | | |
| --- | --- | --- | --- | --- | --- | --- |
| Item | 1 (7.86) | 2 (2.01) | 3 (1.44) | 4 (1.25) | 5 (1.23) | 6 (1.01) |
| 16 | 0.65 | 0.02 | 0.09 | 0.28 | -0.02 | 0.01 |
| 21 | 0.63 | 0.17 | 0.12 | 0.16 | -0.06 | 0.03 |
| 17 | 0.52 | 0.38 | 0.22 | 0.24 | 0.09 | 0.10 |
| 6 | 0.50 | 0.27 | 0.32 | 0.03 | 0.08 | -0.14 |
| 24 | 0.45 | 0.32 | 0.12 | 0.34 | 0.11 | 0.15 |
| 2 | 0.44 | 0.14 | 0.11 | 0.36 | 0.26 | 0.15 |
| 4 | 0.43 | 0.13 | 0.10 | -0.01 | 0.17 | 0.09 |
| 12 | 0.16 | 0.66 | 0.25 | 0.14 | 0.19 | 0.00 |
| 14 | 0.05 | 0.64 | 0.31 | 0.18 | 0.07 | 0.19 |
| 13 | 0.39 | 0.60 | 0.15 | 0.24 | 0.11 | 0.12 |
| 15 | 0.26 | 0.45 | 0.13 | 0.06 | -0.08 | 0.05 |
| 1 | 0.17 | 0.42 | 0.31 | 0.00 | 0.21 | -0.05 |
| 9 | 0.11 | 0.19 | 0.65 | 0.17 | 0.03 | 0.09 |
| 18 | 0.23 | 0.23 | 0.64 | -0.04 | 0.13 | -0.06 |
| 19 | 0.10 | 0.26 | 0.58 | 0.12 | 0.07 | 0.19 |
| 7 | 0.01 | 0.38 | 0.46 | 0.30 | 0.20 | 0.08 |
| 10 | 0.31 | 0.09 | 0.44 | 0.21 | 0.15 | -0.02 |
| 22 | 0.17 | 0.08 | 0.19 | 0.71 | 0.11 | -0.23 |
| 23 | 0.42 | 0.25 | 0.22 | 0.60 | -0.01 | 0.09 |
| 8 | 0.29 | 0.34 | 0.31 | 0.41 | 0.09 | -0.05 |
| 25 | 0.13 | 0.13 | -0.05 | 0.35 | 0.31 | 0.06 |
| 5 | -0.02 | 0.07 | 0.15 | 0.11 | 0.67 | -0.00 |
| 3 | 0.16 | 0.12 | 0.42 | 0.03 | 0.48 | 0.09 |
| 20 | 0.15 | 0.17 | 0.14 | -0.02 | 0.01 | 0.39 |
| 11 | 0.36 | 0.11 | 0.06 | 0.29 | -0.17 | -0.36 |
